# MSRDL: Deep learning framework for service recommendation in mashup creation

**DOI:** 10.1038/s41598-023-32814-y

**Published:** 2023-05-11

**Authors:** Ting Yu, Hailin Liu, Lihua Zhang, Hongbing Liu

**Affiliations:** 1 Jiaxing Nanhu University, Jiaxing, 314001 China; 2 State Grid Jiaxing Electric Power Supply Company, Jiaxing, 314033 China

**Keywords:** Computer science, Information technology

## Abstract

In recent years, service-oriented computing technology has developed rapidly. The growing number of services increases the choice burden of software developers when developing service-based systems, such as mashups or applications. How to recommend appropriate services for developers to create mashups has become a basic problem in service-oriented recommendation systems. To solve this problem, people have proposed various methods to recommend services to match the requirements of the new mashups and achieved great success. However, there are also some challenges in feature utilization and text requirement understanding. Therefore, we propose a Mashup-oriented Service Recommendation framework based on Deep Learning, called MSRDL. A content component was designed in MSRDL to generate the representation of mashups and services. Besides, an interaction component was created in MSRDL to model the invocation records between mashups and services. The output features of the two parts are further integrated into MLP to obtain the service recommendation lists. Experimental results on ProgrammableWeb datasets show that our method is superior to the state-of-the-art methods.

## Introduction

With the increasing popularity of service-oriented computing (SOC)^1^ , more and more developers benefit from reusing web-based services. Using software as a service enables users/developers to integrate software components from different providers and ultimately generate value-added software combinations (such as mashups)^2–4^. Mashup is a developer-centered technology that can combine services to create more comprehensive web applications. In addition, mashups can be published and reused. Mashup development is one of the end user development (EUD) models. It helps IT professionals repurpose existing services and develop integrated applications for themselves. Internet companies such as Microsoft, IBM, and Google have developed several mashup application platforms to authorize end users to create mashups. When using the mashup application platform, the creator operates through an easy-to-use web-based UI. Therefore, the development cycle and cost of applications are greatly reduced. Despite the above convenience, it is still challenging to select the right services for end users in the process of mashup development.

**Figure 1:**
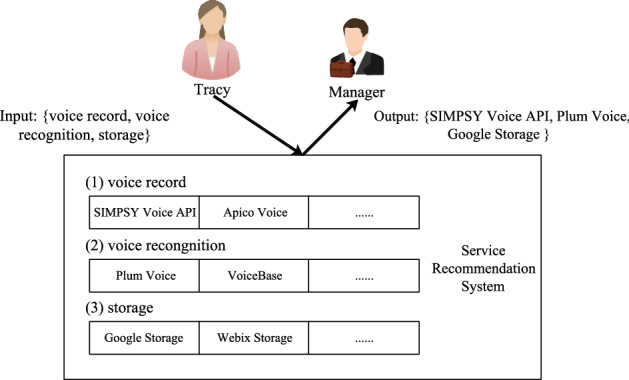
A toy example of the process of service search. The figure is created using ORIGINLAB ver.2023 (https://www.originlab.com/).

Figure 1 shows an example of the process of service search. Tracy, a reporter, is going to interview a business manager. In order to successfully complete this interview, Tracy needs to find a mashup or application that can perform three subtasks, namely voice recording, voice recognition and storage. In this case, Tracy queries the service by entering three function keywords voice record, voice recognition and storage to find the best service list. In this case, several key issues are raised in the service recommender system. First, for Tracy, when she queries the recommendation system, listing three keywords (voice recording, voice recognition and storage) is usually a challenging task, because Tracy is not an expert in the field of service search. The search method of services based solely on keyword matching may ignore high-quality services. For example, even if 0‘speech” and “voice” are synonymous, the Google Cloud Speech API will not appear in the list of candidate services for speech recognition.

In conclusion, the challenges faced by the existing research are as follows:

1. The current service recommendation solution requires software developers to enter several keywords to describe the functions of the mashup to be developed, which usually brings great challenges to the service evaluation process of developers because some developers know little about the functions of the service.
2. Existing studies usually use accurate keyword matching technology for service search, while ignoring possible synonyms and word changes. This disadvantage is easy to narrow the scope of service selection and ignore high-quality services.
3. CF-based methods can share the experience of similar mashups, and also can achieve certain results in the case of sparse data, but do not give enough attention to the matching between services and mashup requirements.

To overcome the limitations of existing recommendation models, this paper proposes a Service Recommendation approach for a Mashup-oriented web Service Recommendation framework based on Deep Learning, called MSRDL. Firstly, Considering that deep learning technology has made great progress in natural language processing^5^, we utilize the content component to extract semantic features of the requirements of the new mashup and services and express it as a semantic representation. Then, the semantic representation of the mashup and service will be input into the fully connected neural network to calculate the similarity between them. Secondly, the interaction component is utilized to learn the interactive information of existing mashups and candidate services. We obtain the Top-N neighbor existing mashups which are similar to the new mashup based on the semantic representation. Based on the neighbors of the new mashup, we can get the preference of the new mashup for services. Finally, we fuse the above two results to obtain the final recommendation. The content component can better capture the characteristics of requirements through a deep understanding of the content, while the interaction component can provide us with the ability of memory-based collaborative filtering. The main contributions of this paper are threefold.

1. The Transformer model is leveraged for the computation of the functional similarity between the mashup requirements and service description. This approach presents the advantage of being capable of comprehending the meaning of sentences, whilst simultaneously circumventing the potential inaccuracies stemming from the weighting of word vectors.
2. Utilizing mashup-service invocation records as the basis, we leverage a LightGCN model to obtain embeddings for both mashups and services. Compared to traditional neural networks, this approach excels in feature extraction.
3. Extensive experiments have been conducted on a real-world dataset to evaluate the proposed approach. The results indicate that the proposed approach achieves better performance compared to the state-of-the-art methods.

The remainder of the paper is organized as follows. After Section 2 discusses the related work, Sect. 3 introduces the approach in detail. Section 4 discusses the comprehensive experimental results on the real world dataset. Finally, Sect. 5 concludes the paper and briefly discusses the future research work.

## Related works

With their great success in commercial application, recommendation systems^6,7^ have attracted great attention from the research circles. Depending on the type of information used in the recommender system, existing service recommendation approaches are mainly divided into three categories: content-based^8,9^, QoS-based^10,11^ and service recommendation with deep learning technology^12,13^.

### Content-based service recommendation

Content-based service recommendation methods focus on mining the relationship between mashups and web service requirements, and directly recommend those web services that are close to the mashup. In recent years, recommendations based on the probabilistic topic model have become increasingly popular.

The underlying service topic features are extracted from the topic model, and service recommendation is made by service topic feature matching. Gao et al.^9^ designed a model called SECO-LDA based on the LDA topic model to mine the potential co-occurrence topic relationship of services, and then predict the future composition pattern of services, in order to help users quickly develop service composition. Li et al.^14^ employed the to model the tag and topic information of mashups and services, thereby calculating the similarity between services and the similarity between mashups. Meanwhile, the invocation time and category information of the services can be used to derived popularity. The factorization machine is used to model the information of multiple dimensions, such as the similarity between mashups and services, the popularity of services, etc., to predict and recommend the services corresponding to the target mashups. Compared with the above works, we use a deep neural network as the modeling basis to build a complete end-to-end service recommendation framework.

### QoS-based service recommendation

Quality of Service (QoS) is the description or measurement of the overall performance of a service, such as availability, response time, throughput, and reliability. Selecting the composite service with the best QoS value is known as the QoS-based service recommendation.

Kwapong et al.^15^ proposed a session level representation method based on multidimensional attention mechanism to enhance the matching degree between user interaction sequences and user intentions, and reduce the impact of noise interaction. Meanwhile, a time-based directional attention mechanism is integrated into the long short-term memory (LSTM) network, to more effectively capture the sequential patterns of interactive sessions and improve the understanding of users’ dynamic preferences. Rangarajan et al.^16^ suggested service selection strategy using code source metrics. Through verification training on code complexity and functionality measurement, service selection can be effectively carried out. Besides, QoS attribute of reliability sets quality standards for service selection, service discovery, and service invocation. Ma et al.^17^ proposed a general collaborative filtering (GCF) method based on a neural network to model the user-service interactions. However, because QoS is dynamic and changes over time, QoS-aware methods may encounter uncertainty.

### Service recommendation with deep learning technology

With the rapid development of deep learning, it has become a trend to deliver neural networks in the service recommendations. To better model Web service description documents and mine the hidden information, for example, the context and the word order, Shi et al.^18^ proposed a service recommendation method based on text extension and depth model, and LSTM model with two attention mechanisms is employed for service recommendation to help select the most appropriate service. Chen et al.^19^ exploited a neural collaborative filtering model which learns user preference based on invocation records and text information between mashups and services. Xiong et al.^20^ combined collaborative filtering and text content, the invocation interaction and function information of mashup and service are integrated into a deep neural network to represent the complex relationship between mashup and service. Xie et al.^21^ proposed a framework based on Generative adversarial network (GAN) for service recommendation. They build a heterogeneous information network (HIN) that utilized mashups, services and their auxiliary information. Furthermore, they constructed mashup-API similarity matrices based on meta-paths with different semantics. Finally, the service recommendation list can be obtained through the adversarial training.

Generally speaking, the existing methods rely heavily on capturing complex interactions between mashups and services, which is unrealistic when developing new mashups from scratch.

Table 1 summarizes the aforementioned methods on service recommendation.

**Table 1:** Summary of the aforementioned methods on service recommendation.

Approaches	Description	Recommended service type	Typical techniques	Limitations
Content-based	Measure functional similarities between the content of services and mashups	Functional similar services	Keyword-based matching Logical semantics-aware	Much efforts are required for manual semantic annotation
QoS-based	Compare the QoS of services with users’ non-functional requirements	Non-functional similar services	CF with the context of mashups and services	QoS of services are usually unavailable
Deep learning-based	Mine the hidden information services and mashups	User preferred services	Composite-service network	The problem of cold-start service recommendation remains a challenging research topic

## Proposed approach

**Figure 2:**
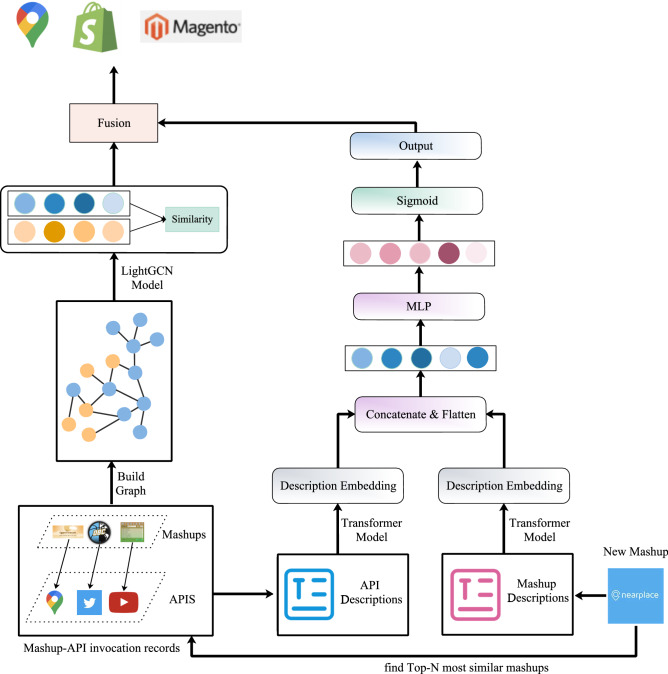
The framework of MSRDL. The figure is created using ORIGINLAB ver.2023 (https://www.originlab.com/).

For service recommendation, there are three steps. Firstly, we build the recommendation model; Secondly, the recommendation model will be trained against the invocation records of services and mashups gathered from ProgrammableWeb. After that, developers or users submit their requirements which described in natural language, and the candidate collection of services will be generated. The framework of this paper is demonstrated in Fig. 2. In this section, we will discuss our method in detail.

### Problem definition

#### Definition 1

(Mashup) A mashup *m* represents the composition of one or many web services into one single application, formalized as a 4-tuple, i.e., $m=<m_n; m_{des};(s_1,s_2...s_n);(m_{t1},m_{t2},...,m_{tj})>$, where $m_n$, $m_{des}$, $(m_{t1},m_{t2},...,m_{tj})$ represent the name, text description and tag sets of *m* and $(s_1,s_2...s_n)$ represents the service record invoked by *m*.

#### Definition 2

(Service) A Service *s* is a structure based on programming language, which makes it easier for developers to create complex functions, formalized as a 3-tuple, i.e., $s=<s_n;s_{des};(s_{t1},s_{t2},...,s_{ti})>$, where $s_n$, $s_{des}$, $(s_{t1},s_{t2},...,s_{ti})$ represent the name, text description and tag sets of *s*, respectively.

#### Definition 3

(Mashup-service invocation) Given a set of mashups as $M =\{m_1,m_2..m_q\}$ and a set of services as $S =\{s_1,s_2...s_p\}$, A Mashup-Service Invocation matrix $Y\in R^{q\times p}$ is defined according to the service invocation by mashups. For mashup m and service s, $y(m,s) = 1$ means that s is invoked by m, and $y(m,s) = 0$ if otherwise.

*Problem definition* Given the mashup service history invocation records and the text information about mashups and services, for a new required mashup at the moment with user queries as a collection of words, a ranked list of services will be recommended to the requesting user. Services with higher rank in the list should have higher probability to be adopted by the user than others with lower ranks.

### Service recommendation

#### Preprocessing

When creating a new mashup $m'$, the developer needs to enter the mashup requirements (represented by $d_i$) consisting of a set of phrases, sentences, or even paragraphs as the developer’s initial query for similar mashups. Such descriptions usually contain noises and cannot be used directly for model learning. To reserve the important information in the texts, the following processes are usually required.

1. Text Filtering: Words such as tags, punctuation marks, non-characters and stop words are usually unmeaningful. The regular expressions can be used to filter them out.
2. Abbreviation Replacement: Abbreviations should be replaced with their complete spellings. For example, ‘dont’ is replaced to ‘do not’.
3. Lemmatization: Lemmatization is the process of converting a word to its base form. The Lemmatization method is based on WorldNet’s built-in morph function. For example, the word “trees” is reduced to the word “tree”, and the word “used” is reduced to “use”.

For example, when developing a mashup named NearPlace as shown in Fig. 2, the description $d_i$ pre-processed to the resulting word set ${d_i}'$= {‘nearplace’, ‘free’, ‘store’, ‘locator’, ‘google’, ‘map’, ‘marker’, ‘product’, ‘user’, ‘friendly’, ‘time’, ‘advanced’, ‘widget’, ‘thanks’, ‘extremely’, ‘easy’, ‘management’, ‘always’, ‘actual’, ‘news’, ‘opening’, ‘hour’, ‘product’, ‘service’, ‘many’}.

#### Content component (CC)

**Figure 3:**
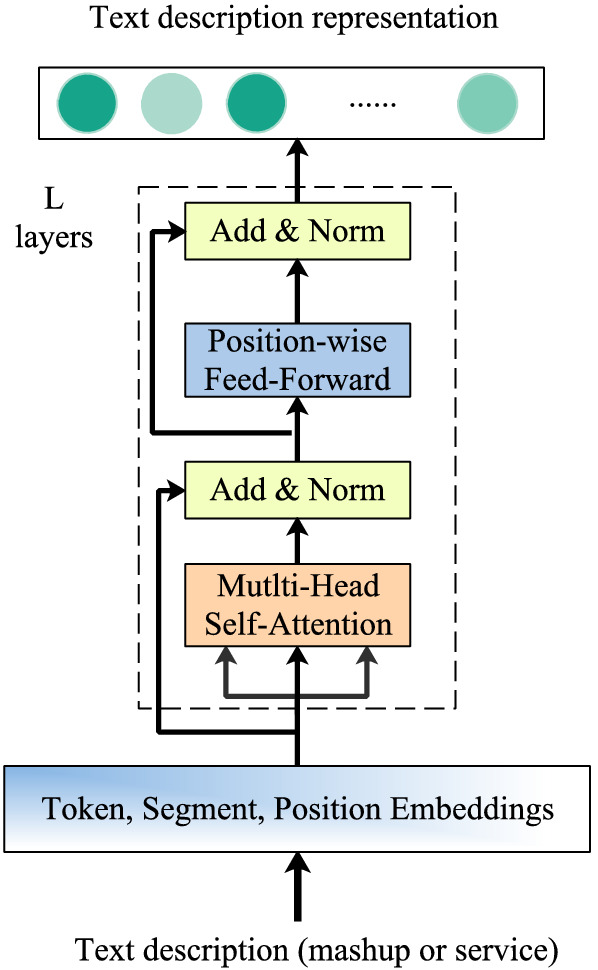
The stage of learning representation of text description.

For a given input token, the corresponding embeddings including three parts, namely token, segment and position. The input representation is constructed by summing these embeddings in our study. Token embedding indicates word embedding of each word, segment segmentation is used to distinguish two sentences, and position embedding refers to encoding the position information of words. A visualization of this construction can be seen in Fig. 3. Transformer uses the task of masking language model for self-supervised training together with the task predicted in the next sentence. Masking language model refers to randomly masking 15% of sub-words in the text, replacing 80% with [MASK] label, 10% with random words, and leaving 10% unchanged. By asking the model to predict the 15% of these sub words that are covered, the model learns the vector representation of the text. The final input token representation $E_t$ in transformer model^22^ is constructed by summing its corresponding token, segment and position embedding as Eq.(1).

1 $$\begin{aligned} E_t=E_{token}+E_{pos}+E_{seg} \end{aligned}$$

As illustrated in Fig. 3, the Word-Level Module (WLM) applies multiple Transformer Layers (TL) iteratively to compute the hidden representations at each layer for each word and propagate the matching signal at word-level simultaneously. Each TL contains two sub-layers: a Multi-Head SelfAttention sub-layer and a Position-wise Feed-Forward network.

The Self Attention can be described as mapping a set of key-value pairs of query and key value to the output, and the output is calculated as the weighted sum of values. The weight assigned to each value is calculated by the similarity function between the query and the corresponding key This form of attention is called Scaled Dot Product Attention, and the corresponding mathematical Equation is as follows:

2 $$\begin{aligned} Attention(Q,K,V)=softmax(\frac{(QK^T)}{\sqrt{d_k}})V \end{aligned}$$

where *Q*, *K* and *V* represents the query, key and value matrix correspondingly, which are projected from $E_t$ matrix with different learned projection matrices as in Eq.(1), and $\frac{1}{\sqrt{d_k}}$ is a scaling factor to avoid extremely small gradients by producing a softer attention distribution. For Multi-Head Self-Attention layers, *Q*, *K* and *V* are first mapped through the parameter matrix, then self-attention is done, and finally the results are concatenated and sent to a fully connected layer. The calculation process is as Eq.(3) and Eq.(4):

3 $$\begin{aligned} head_i=Attention(E_t W_i^Q,E_t W_i^K,E_t W_i^V) \end{aligned}$$

4 $$\begin{aligned} MultiHead(Q,K,V)=[head_1; head_2... head_h]W^O \end{aligned}$$

where $W^O$, $W_i^Q$, $W_i^K$ and $W_i^V$ are learnable parameters, *i* represents $i-th$ head. The experimental results show that multi head can extract the features of different heads at a more detailed level, and the effect of feature extraction is better when the overall computational load is the same as that of a single head. The Position-Wise Feed-Forward network is a fully connected feedforward network, and the words in each position pass through the same feedforward neural network separately. It consists of two linear transformations, that is, two fully connected layers. The activation function of the first fully connected layer is ReLU activation function, can be expressed as Eq.(5) :

5 $$\begin{aligned} FFN(x)=ReLU(xW_1+b_1 ) W_2+b_2 \end{aligned}$$

where $W_1$, $W_2$ and $b_1$, $b_2$ are learnable parameters and shared across all positions. The Transformer layer then employs the residual connection and layer normalization function LN around the above two sub-layers to extract the contextual representation, as Eq.(6) indicates.

6 $$\begin{aligned} v_m=LN(x+FFN(x)) \end{aligned}$$

The final representation of mashup and service, extracted by *L* stacking Transformer layers, are denoted as $v_m$ and $v_s$. We concatenate $v_m$, $v_s$, then utilize an MLP to capture interactions among $v_m$, $v_s$. Moreover, we select the parametric rectified linear unit (ReLU) as the activation function since it can improve model fitting with nearly zero extra computational cost and little overfitting risk. The learning process can be written as Eq.(7):

7 $$\begin{aligned} i_{m,s}=MLP(v_m\oplus v_s) \end{aligned}$$

where $i_(m,s)$ is the learned vector. We finally feed the learned interaction vector into a sigmoid function, whose output, ${\hat{r}}$, represents the probability of *m* selecting *s* as the service to be recommended. The process can be written as Eq.(8) :

8 $$\begin{aligned} {\hat{r}}=sigmoid(W^T i_{m,s}+b) \end{aligned}$$

#### Interaction component (IC)

**Figure 4:**
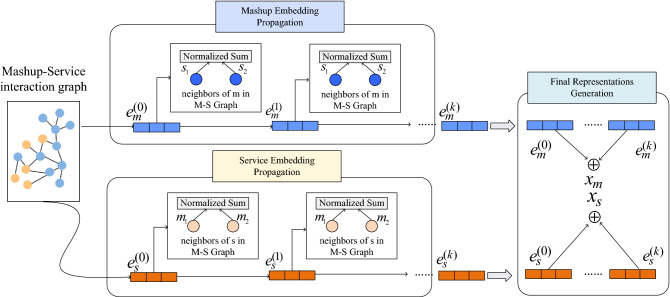
Architecture design of IC. The figure is created using ORIGINLAB ver.2023 (https://www.originlab.com/).

Through the mashup-service innovation matrix, we build a mashup service tag network graph $G = (V,E)$, where $V=\{v_1,v_2...v_n\}$ represents the node set, and *E* represents the edge set. Here, mashup set *M*, service set *S* are used to build the node set $V = M\cup S$. For the edge set *E*, if an invocation between the mashup *m* and service *s*, they are connected in the graph, thus forming the edge set of $E_{m,s}$. The mashup-service network graph is trained based on the LightGCN model^23^ as Fig. 4 shows. It consists of 3 parts: (1) a embedding layer, the mashups and services from their identifiers (IDs) is mapped to a dense vector with fixed dimensions, (2) multiple propagation layers, the embeddings in the mashup-service graph are propagated, and (3) final representations generation, the final representations of the mashups and services are generated by this part.

The embedding layer encoders a mashup *m* (or a service *s*) with a fixed-length vector $e_m^{(0)}\in R^d$ ($e_s^{(0)}\in R^d$). Such a vector is called an embedding. Here *d* is pre-specified parameter, denoting the size of an embedding. The superscript “k” is a layer index, indicating that the embeddings are the output of the $k-th$ propagation layer, the superscript “0” is used to indicate the output of the embedding layer. After being initialized in the embedding layer, the embeddings will be iteratively optimized in the sub-sequential training process of the LightGCN model. For a mashup *m* and a service *s*, we use the mashup-service interaction graph to update the mashup embedding outputted from the ($k-1$)-th layer. To do this, two embeddings are generated for the mashup *m* and service *s* in the mashup-service interaction graph as Eq.(9) and Eq.(10), denoted $x_m^{(k)}$ and $x_s^{(k)}$.

9 $$\begin{aligned} x_m^{(k)}=\sum _{s\in N_m}\frac{1}{\sqrt{\vert N_m\vert } \sqrt{\vert N_s\vert }}x_s^{(k-1)} \end{aligned}$$

10 $$\begin{aligned} x_s^{(k)}=\sum _{m\in N_s}\frac{1}{\sqrt{\vert N_m\vert } \sqrt{\vert N_s\vert }}x_m^{(k-1)} \end{aligned}$$

Where $N_m$ and $N_s$ denote the mashup *m*’s neighborhood set and service *s*’s neighborhood set in mashup-service interaction graph. After being propagated through the *K* layers, the embeddings of the mashup *m* and service *s* are obtained, i.e., $\{x_m^{(0} ), x_m^{(1)}... x_m^{(k)}\}$ and $\{x_s^{(0)},x_s^{(1)} ... x_s^{(k)}\}$ respectively. Based on this, the final representations of the mashup and service are generated by summing up the outputs of the *K* layers Eq.(11) and Eq.(12):

11 $$\begin{aligned} x_m= & {} \sum _{k=0}^Ka_kx_m^{(k)} \end{aligned}$$

12 $$\begin{aligned} x_s= & {} \sum _{k=0}^Ka_kx_s^{(k)} \end{aligned}$$

#### Service selection and ranking

Through the transformer model, we obtain the text requirements vectors of the mashups and services, denoted as $v_m$ and $v_s$ . Meanwhile, by employing the LightGCN model, we obtain the low dimensional representations of the mashup *m* and service *s*, denoted as $x_m$ and $x_s$. When a new mashup $m'$ needs to be created, we calculate its representation vector using Eq.(13):

13 $$\begin{aligned} v_{m'}=\sum _{nm'\in N(m')} cosine(v_{m'}, v_{nm'})\cdot x_{nm'} \end{aligned}$$

where $nm'$ is the neighbor of the new mashup $m'$. In this paper, we select Top-N neighbor mashups that are most similar to the new mashup $m'$ as the final representation. $x_{nm'}$ is the representation of the neighbor mashup obtained by the LightGCN model. The raking score of the mashup *m* on service *s* is calculated by taking the inner product of their representations as Eq.(14):

14 $$\begin{aligned} p_{m'}^s=\frac{\sum _{nm'_{i=1}}^Nx_{nm'_i}\cdot x_s}{N} \end{aligned}$$

where *N* is the number of the neighbor mashups of $m'$, $x_s$ is the embedding of service *s* that obtained by the LightGCN model. Finally, the preference of mashup *m* for service *s* is shown in Eq.(15):

15 $$\begin{aligned} s_{final}={\hat{r}}+p_{m'}^s \end{aligned}$$

## Experiment

The dataset was crawled from ProgrammableWeb, the world largest online Web service registry, on March, 2021. The mashups and services without functional descriptions, the services that have not been invoked were removed from the original dataset. Finally, our dataset includes 7845 mashups, 1709 services which were invoked by mashups at least once. All experiments were implemented in Python and conducted on a personal computer equipped with an Intel Core i5 CPU clocked at 2.4 GHz and 8 GB RAM, running the macOS High Sierra operating system.

### Evaluation metrics

Users can request services by writing and submitting their queries, and then the recommendation system gives them back the top-K most relevant results. We used the following metrics to evaluate recommendation results and averaged the five-folds’ metric values as the final evaluation result. Precision, signed as *Precision*@*k*, measures how many recommended services correspond to the true services in testing data for a given mashup, as shown in Eq.(16), where $top_m(k)$ represents the top *k* services recommended to mashup *m*, and $test_m$ represents the services actually invoked by mashup *m* in the test set. The precision of the model represented by $Precisio-n@k$ is the average precision of all mashups. Recall, signed as *Recall*@*k*, measures how many true services in testing data have been recommended for a given mashup as shown in Eq.(17). The recall of the model represented by *Recall*@*k* is the average recall of all mashups. The Mean Average Precision (MAP) at top *k* services in the ranking list is defined as Eq.(18). where *I*(*i*) indicates whether a service at the position *i* in the recommendation ranking list is an actual component service, $k_m$ is the number of component services of mashup *m*, and $k_i$ denotes the number of actual component services of the mashup occurring in the top *i* services of the recommendation ranking list.

16 $$\begin{aligned} Precision@k= & {} \frac{1}{n}\sum _{m=1}^n\frac{\vert top_m(k)\cap test_m\vert }{k} \end{aligned}$$

17 $$\begin{aligned} Recall@k= & {} \frac{1}{n}\sum _{m=1}^n\frac{\vert top_m(k)\cap test_m\vert }{\vert test_m\vert } \end{aligned}$$

18 $$\begin{aligned} MAP@k= & {} \frac{1}{n}\sum _{m=1}^n\frac{1}{k_m}\sum _{i=1}^k(\frac{k_i}{i}\times I(i)) \end{aligned}$$

### Evaluation methods

We compare MSRDL with the following strong baselines that are applied for the service recommendation field:

- Pop: It first calculates the number of times each service is invoked in the mashup and ranks them based on their count or popularity. It then recommends the Top-K popular service for each mashup.
- TF-IDF: It recommends services whose descriptions are similar to that of the target mashup based on vector space model. The term frequency and inverse document frequency are used to calculate the cosine similarity between the services and the target mashup.
- SFTN^24^: It uses topic models and neighbor interaction probabilities to calculate similarity scores between services and requirements, then multiply these scores to rank candidate services.
- NGCF^25^: It first uses the transformer model to represent the word embeddings of mashup and service descriptions, then employs NGCF to explicitly construct a bipartite mashup-service graph to model the high-order connectivity.

### Performance

**Figure 5:**
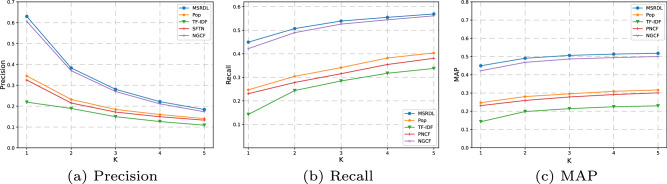
Performance comparison of different approaches.

We finely tuned the parameters for all comparison methods, and compared their performance based on the well-tuned parameters for the sake of fair comparisons. Basically, we set the parameters of comparison methods to the values given by the corresponding researchers, which can be regarded as the best ones. More specially, for the service recommendation, we set the parameters for SFTN as in^24^,for NGCF as in^25^ and for LIGHTGCN as in^23^. As shown in Fig. 5, we found that TF-IDF performs the worst, even though it uses text description. The reason may lie in that it ignores the order of words and further leads to lost semantic information. In addition to descriptive information and popularity, SFTN handles historical usage information somewhat better, but the poor service/requirement representations obtained by the topic model remain limited to its performance. Simple as pop is, it performs slightly worse than SFTN. NGCF performs fairly well. The possible reason is that it extracts high quality features from the content information and learns the deep interaction between mashups and services like MSRDL. Compared with all the baseline models, MSRDL has the advantages of both CF-based methods and content-based methods. MSRDL outperforms them significantly. The trend is more significant when Top-1 recommendation is considered. Because each mashup uses only a small number of services, averaging less than two, it makes sense to look at the head of the recommendation list. As N ranges from1 to 5, MSRDL improves the baseline methods by at least 5.7%-72.9% in Precision, 6.1%-76.7% in Recall and 6.1%-76.7% in MAP.

### Ablation study

**Figure 6:**
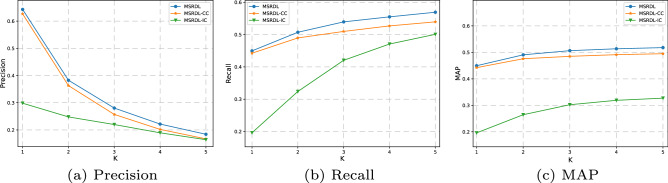
Performance of MSRDL variants.

Ablation study is widely used to detect the importance of components in machine learning models, especially in complex neural networks. For our MSRDL, there are two components worth studying and analyzing. The first one is the IC which provides interactive recording for MSRDL. By removing it from MSRDL, we obtained an ablation model as the MSRDL-IC. The second one is CC which provides requirements extraction for MSRDL. By removing it from MSRDL, we obtained an ablation model as the MSRDL-CC. Figure 6 shows the performance comparison of MSRDL, MSRDL-IC, and MSRDL-CC on the task of service recommendation. By comparing MSRDL, MSRDL-IC and MSRDL-CC in Fig. 6, we find that adding the requirements extraction can bring 2.5%-9.2% of the revenue in Precision@1-5,1.6%-5.4% of the revenue in Recall@ 1-5 and 1.6%-4.4% of the revenue in MAP@ 1-5. we find that adding the interactive recording can bring 49.9%-95.9% of the revenue in Precision@1-5, 89.8%-96.4% of the revenue in Recall@1-5 and 91.5%-96.4% of the revenue in MAP@ 1-5.

### Parameter impact

**Figure 7:**
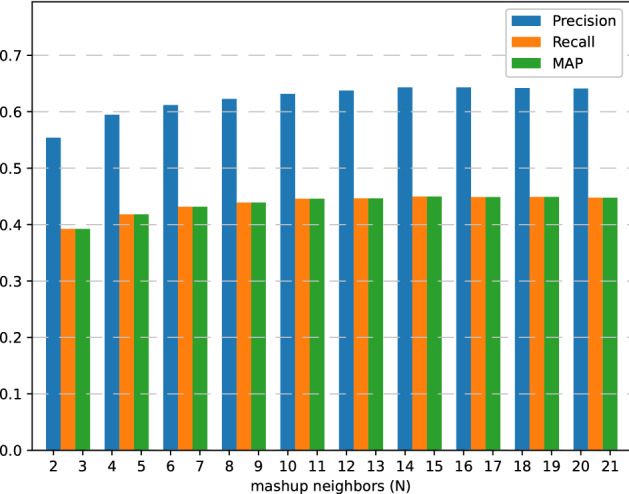
Architecture design of IC.

As shown in Fig. 7, when *N* increases from 2 to 20, the performance of MSRDL increases. This result may be the increase in the size of neighbor mashups helps MSRDL learn more about the invocation history of similar mashups. Nevertheless, when *N* exceeds the critical point, the recommended performance of MSRDL becomes worse. This may be due to the introduction of some noise data in the learning of neighbor interaction, i.e., the mashups with low similarity to the target mashups.

### Running time comparison

**Figure 8:**
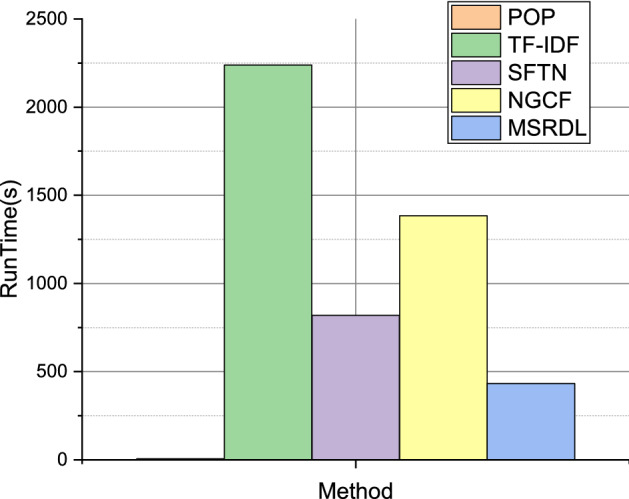
The running time of the MSRDL compared with the baseline methods.

Figure 8 reveals that the POP method outperforms all other methods in terms of speed, completing the recommendation task within a few seconds. However, its recommendation performance is suboptimal. The MSRDL method we proposed shows competitive performance in terms of recommendation accuracy, albeit with a longer processing time. Specifically, TF-IDF incurs the highest processing time, followed by NGCF and SFTN. In view of the effectiveness and efficiency trade-off, our proposed MSRDL method achieves superior performance. It follows that, compared with related deep learning-based methods, the empirical running time of our method can be deemed to be relatively acceptable.

## Conclusion

To solve the problem of service selection during mashup creation, we propose a Mashup-oriented Service Recommendation framework based on Deep Learning model, called MSRDL. In MSRDL, we propose two components: Content Component and Interaction Component. Content Component fully exploits description information, while Interaction Component fully exploits mashup service invocation records. Finally, we effectively integrate the two together to make the final service selection. In the future, we plan to incorporate QoS attributes into the service recommendation process. Secondly, a more powerful neural network model can be used in the MSRDL model to enhance the effect of description modeling.

## Data Availability

The datasets generated and/or analyzed during the current study are available in the ProgrammableWeb repository, .
